# Prevalence of Arterial Hypertension in Cirrhosis of Liver

**DOI:** 10.4103/1319-3767.45067

**Published:** 2009-01

**Authors:** Rajesh Prabhu P., Srinivasan R., Jayanthi V.

**Affiliations:** Department of Gastroenterology, Stanley Medical College, Chennai, India; 1 Heavy Vehicles Factory – Health Unit, Avadi, Chennai, India. E-mail: p.rajeshprabhu@gmail.com

Sir,

Systemic hypertension is a common disorder. Cirrhotic patients have hyperdynamic circulation with a decrease in effective circulatory blood volume, which has been attributed to the underlying vasodilatory state in these patients.

On this background, we conducted a study to evaluate the prevalence of essential hypertension among patients with cirrhosis and the related cardiovascular changes among them. We prospectively evaluated 260 cirrhotic patients at the Stanley Medical College, Chennai, India, regularly attending the Liver Clinic, of whom alcohol was the cause in 140 cases. All patients underwent blood pressure (BP) recording in supine and sitting posture, ECG, and echocardiogram. Among the 260 cases, 23 (8.8%) patients with arterial hypertension were identified, of whom four patients were excluded because of secondary hypertension. Among the remaining 19 (7%) patients with hypertension, the mean age was 51 ± 14.46 years, M:F ratio was 3.1:1, and the mean BMI was 25.8 kg/m^2^. The mean arterial pressure (MAP) was 120 mmHg. Ten patients (52.6%) were categorized as Child's A, six (31.5%) as Child's B, and three (15.7%) as Child's C. Abnormalities in ECG were noted in seven patients (36.8%), and abnormalities in echocardiogram were observed in six (31.6%) patients. In the normotensive group, comprising 237 patients, the mean age was 53 ± 13.56 years, M:F ratio was 1.8:1, mean BMI was 24.2 kg/m^2^, and the MAP was 83 mmHg; abnormalities in ECG were present in four (1.6%) patients, and none had abnormalities in echocardiogram.

There has been a relatively low prevalence of hypertension among patients with hepatic cirrhosis.[1] Cirrhosis is a state of hyperdynamic circulation with increased cardiac output and low systemic vascular resistance.[2] This is mainly due to the abnormal distribution of increased blood volume with decreased effective circulatory blood volume, neurohumoral activation, and abnormal sodium and water handling.[3] There is also evidence that the static and dynamic properties of the large arteries are changed.[4] These factors probably account for the relatively low incidence of hypertension in patients with cirrhosis.

In the current study, the prevalence of hypertension among cirrhotic patients was 7%, which is less than that among the general population (10–15%). Incidence of hypertension was found to be higher in patients with Child's A cirrhosis and showed a decreasing trend on worsening of liver disease. The study has shown the relatively low incidence of hypertension and cardiac events among the patients with cirrhosis, thereby postulating the possible protective effects of cirrhosis on the cardiovascular system.
